# Racialized Structural Vulnerability: Neighborhood Racial Composition, Concentrated Disadvantage, and Fine Particulate Matter in California

**DOI:** 10.3390/ijerph16173196

**Published:** 2019-09-01

**Authors:** Raoul S. Liévanos

**Affiliations:** Department of Sociology, University of Oregon, Eugene, OR 97403-1291, USA; raoull@uoregon.edu

**Keywords:** particulate matter, race, segregation, environmental inequality, population vulnerability, spatial analysis, California, CalEnviroScreen

## Abstract

This study contributes to previous research by advancing a “racialized structural vulnerability” framework and presenting a new empirical analysis of the relationship between neighborhood Asian, Black, and Latinx composition; extrinsic and intrinsic vulnerability; and PM_2.5_ exposures in California with secondary data from 2004–2014. Principal component analyses revealed that tract Latinx composition was highly correlated with extrinsic vulnerability (economic disadvantage and limited English-speaking ability), and that tract Black composition was highly correlated with intrinsic vulnerability (elevated prevalence of asthma-related emergency department visits and low birth weight). Spatial lag regression models tested hypotheses regarding the association between Asian, Black, and Latinx population vulnerability factors and the 2009–2011 annual average PM_2.5_ percentile rankings, net of emissions and spatial covariates. Results indicated that the percent Latinx population, followed by the regional clustering of PM_2.5_, and the percent of non-Latinx Black and non-Latinx Asian population were the strongest positive multivariable correlates of PM_2.5_ percentile rankings, net of other factors. Additional analyses suggested that despite shifting demographic and spatial correlates of 2012–2014 PM_2.5_ exposures, the tracts’ Black and Latinx composition and location in the San Joaquin Valley remain important vulnerability factors with implications for future research and policy.

## 1. Introduction

### 1.1. Background

Airborne fine particulate matter (PM_2.5_) is a complex chemical mixture of solid and liquid particles that are emitted directly into the air or result indirectly from the conversion of precursor chemicals. PM_2.5_ has an aerodynamic diameter less than 2.5 μm, which is significantly smaller than the average diameter of a human hair (~70 μm) and fine beach sand (~90 μm) [1]. PM_2.5_ can remain airborne for days to weeks and travel as far as 1000 km from its natural and anthropogenic sources—the latter of which are primarily traffic and industrial emissions [2]. These attributes of PM_2.5_ enable it to travel great distances and penetrate critical gas exchange activity in the lungs as well as lung tissue and even the blood stream [3]. Around the world, elevated PM_2.5_ exposures contribute to premature death, adverse fetal and infant health outcomes, and a variety of cardiovascular and respiratory impairments and diseases [1,2,4].

Research outside the United States indicates that PM_2.5_ concentrations and exposures vary significantly across time, space, and population subgroups. For example, PM_2.5_ concentrations reach hazardous levels throughout the year across Delhi, India, but they spatially cluster in select areas of that mega city from November to March [5]. A survey-based, time-activity pattern study in Seoul, South Korea found that younger individuals, those working longer hours, and those working outside office settings had elevated PM_2.5_ exposures during the summer [6].

Studies within the United States tend to focus on the extent to which PM_2.5_ concentrations and exposure patterns reflect the nation’s broader social problems of racial inequalities in exposure to environmental health threats [7,8,9,10,11]. For example, one neighborhood-level study that covered the US Midwest, South, and Northeast regions found that average PM_2.5_ concentrations from 2002 to 2006 were consistently elevated in densely-settled (urban) areas with high concentrations of Blacks and levels of racial isolation [12]. Another study analyzed the relationship between individual racial status (White, Black, Hispanic, and Chinese), neighborhood racial composition and segregation patterns, and household-level exposure to average PM_2.5_ levels in 2000 within six cities across the United States [13]. Noteworthy among its results, the study found that residence in primarily White neighborhoods correlated with lower PM_2.5_ exposures, while residence in primarily Hispanic neighborhoods correlated with higher PM_2.5_ exposures. More recently, a nationwide, longitudinal, multi-level study found that, when compared to Whites, Blacks and Hispanics/Latinos (hereafter referred to with the gender-neutral term, “Latinxs” [14]) experienced significantly higher levels of exposure to PM_2.5_ concentrations from 1990 to 2009 in their census block of residence, net of other individual- and metropolitan-level factors [15].

### 1.2. The California Case and Research Question

The present study contributes to previous research with a new statewide analysis of the relationship between neighborhood-level Asian, Black, and Latinx composition; extrinsic and intrinsic vulnerability; and PM_2.5_ exposures in California, USA. California may be understood as an “extreme case” [16] among other United States for its excessive PM_2.5_ concentrations. After the United States Environmental Protection Agency (US EPA) lowered the PM_2.5_ annual primary National Ambient Air Quality Standard (NAAQS) from 15 μm/m^3^ to 12 μm/m^3^ in 2012, it projected using 2009 to 2011 monitoring data that only seven US counties would exceed that new standard by the 2020 target implementation date [17]. Importantly, those counties are located in Southern California (Imperial, Los Angeles, Riverside, and San Bernardino Counties) and California’s San Joaquin Valley (Kern, Merced, and Tulare Counties). Recent research using version 2.0 of the California Community Environmental Health Screening Tool (CalEnviroScreen) elaborates on these points by demonstrating that cumulative pollution burden from air-, land-, and water-based environmental pollutants was significantly concentrated in these regions of California [18]. In addition, that study found that PM_2.5_ is the major environmental health hazard contributor to the unequal spatial distribution of cumulative pollution burden in the state [18].

While California has excessive PM_2.5_ levels, research on racial inequalities in PM_2.5_ exposures throughout California is limited and at times inconsistent with other US-based studies [7,8,9,10,11,12,13,14,15]. One influential California-based study analyzed data from CalEnviroScreen 1.1 and 2010 decennial census data on racial composition at the zip code level, while controlling for population density [19]. That study found that Latinxs, Blacks, Indigenous peoples, Asian/Pacific Islanders, other Nonwhites, and multiracial individuals were significantly more likely than non-Latinx Whites to live in areas with a higher cumulative pollution burden, but found no significant racial disparities in the distribution of PM_2.5_ pollution burdens. This finding suggests that annual mean concentrations of PM_2.5_ are not significantly associated with aggregate-level racial composition in California. A recent analysis of CalEnviroScreen 3.0 found that PM_2.5_ concentrations from 2012 to 2014 had moderate to low positive correlations with various population vulnerability indicators in CalEnviroScreen [20]. However, that analysis, along with an earlier pilot study of proposed CalEnviroScreen inputs [21], did not consider aggregate-level racial composition as a population vulnerability factor because CalEnviroScreen omits racial variables. The omission of racial composition indicators in CalEnviroScreen is an apparent effort to make the screening tool more widely applicable to a diverse range of regulatory decision processes that are prohibited from considering racial factors [18].

Studies of the hazardous Southern California region attend to racial disparities in PM_2.5_ exposures and are consistent with the broader literature on race-based environmental inequality outcomes within that region [22,23] and in the United States [7,8,9,10,11,12,13,14,15]. In Los Angeles County, for example, Blacks, Asian/Pacific Islanders, Latinxs, and other Nonwhites have been found to experience elevated exposures to PM_2.5_ at the census tract level [24] and parcel level [25]. Two related studies analyzed the relationship between modeled ambient air pollutant concentrations in 1998 and travel survey data from 2000 to 2002 for over 25,000 individuals living in the South Coast Air Basin (Imperial, Los Angeles, Orange, Riverside, San Bernardino, and Ventura Counties). Collectively, these studies found that the estimated median intake rates for hexavalent chromium PM_2.5_ and diesel PM_2.5_ were higher for Nonwhites (and low-income households) when compared to the rest of the population studied [26], and when controlling for the individuals’ travel distance, age, sex, education, as well as census tract population density [27].

Previous research on racial inequalities in PM_2.5_ exposures in California is thus limited in its statewide scope and at times inconsistent with other studies that document the elevated PM_2.5_ exposures for Asians, Blacks, and Latinxs in the United States. Accordingly, the present study asks the following research question: In California, what is the relationship between PM_2.5_ exposures; Asian, Black, and Latinx neighborhood composition; other indicators of population vulnerability; industrial and transportation emission factors; and spatial context? This question is addressed through a census tract-level analysis of PM_2.5_ exposures in California with CalEnviroScreen 2.0 and 3.0 as well as additional secondary data on racial composition, industrial land use, and tract location in California air basins. CalEnviroScreen 2.0 data is primarily used throughout the study in order to maintain comparability with recent research that has used CalEnviroScreen 2.0 and found that PM_2.5_ concentrations from 2009 to 2011 significantly contributed to cumulative pollution burden in California [18]. In addition, using the 2009–2011 PM_2.5_ data helped to align this study temporally with the 2010 Decennial Census and 2008–2012 American Community Survey data featured in this study. Likewise, the 2009–2011 PM_2.5_ data aligns with the 2009–2011 air monitoring data used by the US EPA to project non-compliance with the PM_2.5_ annual primary NAAQS of 12 μm/m^3^ by 2020 in the seven Californian counties [17]. CalEnviroScreen 3.0 was used with additional analyses in the discussion section of the article to draw out future research and policy implications from the main variables considered in the analysis from CalEnviroScreen 2.0, the 2010 Decennial Census, and California data on air basins and industrial land use. 

### 1.3. Conceptual Framework and Hypotheses

In this study, an environmental health and inequality conceptual framework featured in a recent study of cumulative pollution burden in California with CalEnviroScreen 2.0 was adapted [18]. That study used multivariable spatial regression techniques to model census tract-level variation in the elevated cumulative pollution burden as a linear function of elevated population vulnerability, emission sources, and spatial dynamics. The population vulnerability component of the framework incorporates the broader notion that “intrinsic vulnerability” (i.e., health conditions) and “extrinsic vulnerability” (i.e., economic status and linguistic ability) are associated with—and can exacerbate the effects of—unequal exposure to environmental stressors [28]. Consistent with studies of race-based environmental health vulnerabilities, that study conceptualized tract racial composition as a component of extrinsic vulnerability [7,8,9,10,11], where it was found that the tracts’ Black and Latinx composition were each positively correlated with cumulative pollution burden. However, its regression analysis found that the Latinx composition was the strongest net demographic multivariable correlate of cumulative pollution burden [18].

**Figure 1:**
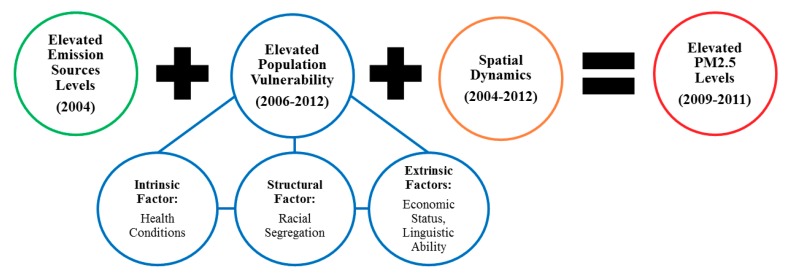
Racialized structural vulnerability framework for modeling population vulnerability and the multivariable correlates of PM_2.5_ levels in California.

Figure 1 visualizes the present study’s “racialized structural vulnerability” framework for analyzing PM_2.5_ exposures. It builds on the “community-level vulnerability” dimensions of Gee and Payne-Sturges’s [9] environmental health disparities framework, which attributes race-based environmental health vulnerabilities to the “structural” factor of racial segregation. Sociologists such as W.E.B. Du Bois [29,30,31] and Cedric Robinson [32], as well as more recent formulations by critical race and environmental justice theorists, have deepened our understanding of how race-based environmental health vulnerabilities are rooted in historical and ongoing effects of racial segregation *and* White supremacy. Collectively, this body of work sees the contemporary US environmental racial formation founded on the erasure of Indigenous peoples, and the subsequent devaluation and segregation of Black, Latinx, and Asian bodies who are associated with filth, waste, uncleanliness, and logical deposits for environmental toxins in urban-industrial spaces [33,34,35]. Meanwhile, White bodies and homogenous spaces are generally associated with purity, cleanliness, and relative environmental privilege away from urban-industrial spaces [36,37,38,39,40]. This environmental racial formation has firm roots in California. For example, historical-comparative analyses reveal that the origins of White supremacy for European Americans in the state prior to 1900 rest in the dispossession of Indigenous peoples “and Mexicans and the economic restrictions imposed on [B]lack, Chinese, and Japanese immigrant workers” [41] (p. 6). Additional research on racial hierarchies in California demonstrates how in subsequent years, institutionalized notions of Nonwhite inferiority and foreignness were deployed by White elites to privilege Whites and suppress Blacks, Latinxs, and Asians to subordinate social, political, and economic statuses, and heightened environmental health hazard exposures [34,36,38,39,42,43,44,45].

Analyses of neighborhood racial composition in California must be placed in this historical context and connected to contemporary manifestations of racial segregation, which operates as a distinct, “structural factor” within the realm of population vulnerability and environmental health disparities [9]. Indeed, a considerable body of sociological theory and empirical research motivate a structural vulnerability notion of racialized population vulnerability that is separate from intrinsic and extrinsic vulnerability in ways that compliment historical and critical race, space, and environmental justice studies frameworks. First, racial identity, classification, and inequalities are not reducible to other aspects of social life [46,47]. Second, critical race and space scholars show how racial segregation is institutionalized and continues to operate in housing markets within and beyond California through such things as discriminatory government and school district policies, mortgage lending, real estate steering practices, and consumer preferences [36,38,40,44,48,49,50,51]. Third, in California, Blacks and Latinxs are independently and differentially burdened with adverse health outcomes like asthma and low birth weight as well as lower economic standing and English-speaking ability [14,18,52,53]. Finally, Blacks, Latinxs, and Asians in some areas of California and throughout the United States continue to experience elevated levels of segregation and exposure to environmental health hazards (including PM_2.5_) relative to other racial groups. Furthermore, these effects endure even when adjusting for other vulnerable social statuses among different racial groups [10,11,12,13,15,18,24,25,54,55,56,57].

The conceptual framework guiding this study motivates three initial hypotheses. They pertain to the heightened vulnerability of exposure to PM_2.5_ among neighborhoods with elevated concentrations of Asians, Blacks, and Latinxs that are linked to institutionalized patterns of racial segregation in California:

**Hypothesis 1 (H1)**.  *The Asian environmental inequality hypothesis states that the spatial concentration of Asians will be positively associated with PM_2.5_ levels, net of other population vulnerability, emissions, and spatial factors.*

**Hypothesis 2 (H2)**.  *The Black environmental inequality hypothesis states that the spatial concentration of Blacks will be positively associated with PM_2.5_ levels, net of other population vulnerability, emissions, and spatial factors.*

**Hypothesis 3 (H3)**.  *The Latinx environmental inequality hypothesis states that the spatial concentration of Latinxs will be positively associated with PM_2.5_ levels, net of other population vulnerability, emissions, and spatial factors.*

This study tests two additional hypotheses drawn from its conceptual framework and previous research in California [18] that pertain to the heightened vulnerability of exposure to PM_2.5_ among segregated Blacks and Latinxs that further concentrate disadvantage (i.e., elevated levels of intrinsic and extrinsic vulnerability) in their neighborhoods of residence:

**Hypothesis 4 (H4)**.  *The Black concentrated disadvantage hypothesis states that the spatial concentration of Blacks and intrinsic and/or extrinsic vulnerability will be positively associated with PM_2.5_ levels, net of emissions and spatial factors.*

**Hypothesis 5 (H5)**.  *The Latinx concentrated disadvantage hypothesis states that the spatial concentration of Latinxs and intrinsic and/or extrinsic vulnerability will be positively associated with PM_2.5_ levels, net of emissions and spatial factors.*

## 2. Materials and Methods

### 2.1. Unit of Analysis

All data analyzed in this study were standardized onto 2010 census tract boundaries. These units provide less precise estimates of human residential settlement patterns and environmental health hazard exposure than smaller census units [54]. However, tracts typically represent locally defined neighborhoods, and they have more reliable population and housing estimates from the US Census and the American Community Survey than smaller census units. Furthermore, tracts are the unit of analysis for CalEnviroScreen 2.0, which is where the majority of the data analyzed in this study were derived.

### 2.2. Dependent Variable 

The dependent variable used in this study is the statewide *percentile of annual average concentration of PM _2.5_ from 2009 to 2011*, as found in CalEnviroScreen 2.0. The averages are quarterly mean concentrations. These were estimated by California state regulatory scientists at census tract centroids using the geostatistical method, ordinary kriging, which incorporates data from nearby monitors in the extensive California Air Resources Board air-monitoring network. Annual means were derived by averaging the quarterly estimates then averaging those over the 2009–2011 period. The statewide distribution of annual average PM_2.5_ concentrations were used to assign a percentile ranking to each census tract. Ninety-one of the 8035 tracts (1.13 percent) included in the dataset had missing PM_2.5_ percentile rankings because their census tract centroids were greater than 50 km from the nearest monitor [58]. One caveat about this variable is that percentile rankings limit one’s ability to distinguish absolute magnitudes of difference between tract-level PM_2.5_ concentrations. However, percentile rankings are central scoring techniques in CalEnviroScreen 2.0 for stratifying tracts according to relative pollution burden [18,58]. Furthermore, percentiles of annual average concentrations of PM_2.5_ approximate a normal distribution when compared to the raw annual average concentration of PM_2.5_, which is a desirable statistical property for the analytical techniques used in this study.

### 2.3. Explanatory Variables: Race-Based Population Vulnerability

The 2010 Decennial Census data on tract racial composition were used to test this study’s five guiding hypotheses. The Asian environmental inequality (H1), Black environmental inequality (H2) and Latinx environmental inequality (H3) hypotheses were respectively tested with the *percent of non-Latinx Asian population*, *percent non-Latinx Black population*, and *percent Latinx population*. Before doing so, it was verified that these racial composition measures captured vulnerable tract racial statuses by assessing bivariate correlations between the percentile of annual average PM_2.5_ concentrations and the percent of tract population that identified as Latinx and non-Latinx White, Black, American Indian/Alaska Native, Asian, Pacific Islander, other Nonwhite racial classification, or multiracial. These initial analyses indicated that out of 7933 tracts with non-missing data, the percentile rankings of annual average PM_2.5_ concentrations for 2009–2011 were only positively correlated with the percent non-Latinx Asian population (Pearson *r* = 0.024; *p* < 0.05), percent non-Latinx Black population (Pearson *r* = 0.101; *p* < 0.001), and the percent Latinx population (Pearson *r* = 0.409; *p* < 0.001).

The Black concentrated disadvantage (H4) and Latinx concentrated disadvantage (H5) hypotheses were tested with measures derived from principal component analysis (PCA). PCA helps to address problems of collinearity between highly correlated variables while facilitating the development of complex and multifaceted indicators of population vulnerability in studies of environmental health and inequality [10,11,12,14,18,59,60]. The PCA techniques used in a previous study of cumulative pollution burden in California with CalEnviroScreen 2.0 were adapted, which used *two separate PCAs* to create respective composite Black and Latinx “cumulative disadvantage” population vulnerability factor variables in testing the hypotheses that were analogous to those found in the present study (i.e., H4 and H5) [18]. The two PCAs used in that prior research extracted unrotated factor solutions through an assessment of the correlations between each component variable by using a maximum of 25 iterations, eigenvalues greater than 1.00, and listwise deletion for missing values. Those two PCAs also featured a regression method to derive a standardized linear combination of factor loading weights for each component variable into separate composite factor scores for Black and Latinx cumulative disadvantage.

The present study draws on PCA techniques found in other studies of CalEnviroScreen [20] and population vulnerability [56] to develop alternative measures of Black and Latinx “concentrated disadvantage.” In doing so, it aimed to obtain a better sense of the multivariate relationships among racial composition and CalEnviroScreen population vulnerability indicators [20]. That is, this study used a *single PCA* that included Black and Latinx composition as well as four indicators of extrinsic vulnerability and two indicators of intrinsic vulnerability within CalEnviroScreen 2.0. Eigenvalues greater than 1.00 and listwise deletion for missing values were used as in prior research [18]. However, in the present study, a varimax rotation was used instead, which limited the extent of variables with high factor loadings, maximized the percent of variation between the factors, and ultimately produced uncorrelated factor components [60]. This alternative technique more clearly delineates how tract Black and Latinx composition are differentially associated with extrinsic and intrinsic vulnerability than was done in prior research [18].

Table 1 displays the inputs used in that analysis. The extrinsic vulnerability indicators used in the PCA were linguistic isolation, low educational attainment, unemployment, and poverty. In California and throughout the United States, these indicators are highly correlated with the presence of Nonwhites, especially of Latinx populations, and are established extrinsic vulnerability factors for environmental health hazard exposure [10,11,14,18,28,59]. Linguistic isolation refers to the percentage of households where no individual of at least 14 years of age speaks English “very well” or speaks English only. The percentage of tract population over 25 years of age with less than a high school education represents low educational attainment. The tract unemployment rate refers to the percentage of tract population over 16 years that was unemployed and eligible for the labor force. CalEnviroScreen 2.0 measures poverty as the percent of tract population whose income in the previous year was below two times the federal poverty level, for example, a single household income of $21,000 a year [18]. These extrinsic vulnerability indicators were derived from five-year average estimates from the 2008 to 2012 American Community Survey (ACS). CalEnviroScreen 2.0 deems ACS estimates reliable if their standard errors are less than half the estimate or less than the mean standard error for all California census tract indicator estimates [58].

Following previous research [18,28,61], two health status indicators of intrinsic vulnerability in the PCA were used. The first of which was a spatially modeled, age-adjusted rate of emergency department visits for asthma per 10,000 people averaged from 2007 to 2009. The rate was initially calculated in five-year age groups, then spatially modeled with a Bayesian technique at the zip code level. It was then reapportioned to census tracts in CalEnviroScreen 2.0 via the areal apportionment of blocks within a tract that intersected those zip codes, and the population-weighted averages of census block rates within a given tract [58]. Percentage of low-weight births (LWB) was the other intrinsic vulnerability indicator and derived from Californian birth records on the extent of live, singleton births from 2006 to 2009 that weighed less than 2500 g. Births were geocoded with the mothers’ residential addresses at birth. LWB rates were spatially modeled to census tracts with an empirical Bayesian technique wherein the number of births in an area set the confidence intervals for the observed LWB rate. That is, few births in an area signified low confidence in the LWB estimate and justified moving the estimate toward the statewide average, while many births in an area indicated high confidence in the LWB estimate and justified little change to that estimate [58].

Two component factors resulted from this PCA, together explaining 68.99 percent of the variance among 7610 census tracts included in the analysis. The first component, *isolated Latinx economic disadvantage*, explained most of the variance among the tracts (42.76 percent) and had a higher eigenvalue (3.421). It is named as such because it loaded high on Latinx composition, linguistic isolation, limited educational attainment, and poverty. The second component, *Black health disadvantage*, explains the remaining variance (26.23 percent) and had a lower eigenvalue (2.098). Its name reflects its high loadings for non-Latinx Black composition, age-adjusted asthma-related emergency department visits, and percent LWB. Black health disadvantage was used in testing the Black concentrated disadvantage hypothesis (H4) and isolated Latinx economic disadvantage in testing the Latinx concentrated disadvantage hypothesis (H5).

### 2.4. CalEnviroScreen 2.0 Extrinsic and Intrinsic Vulnerability Covariates

Consistent with prior research [18], additional measures of extrinsic and intrinsic vulnerability were included from CalEnviroScreen in testing the Black and Latinx environmental inequality hypotheses. However, this was done by applying the PCA techniques discussed above in an additional PCA of the six CalEnviroScreen 2.0 extrinsic and intrinsic vulnerability indicators. Table 2 summarizes the results for the two relatively independent component factors produced from this PCA. Combined, these components explained 71.10 percent of the variance among the 7610 census tracts included in the analysis. The first component, *isolated economic disadvantage*, explained most of the variance (52.81 percent) and had a higher eigenvalue (3.169). It loaded high on linguistic isolation, limited educational attainment, and poverty. The second component, *health disadvantage*, loaded highest on age-adjusted asthma-related emergency department visits and percent LWB. This explains the remaining variance (18.29 percent) and had the lowest eigenvalue (1.098) among all of the factors used in this study.

### 2.5. Emission Sources

Traffic and industrial emission sources are major contributors to airborne PM_2.5_ [2]. These emission sources were accounted for in this study with the same temporally matched measures that were previously found to be positively and significantly associated with cumulative pollution burden in California [18]. Specifically, I operationalized traffic emission sources with the 2004 estimates of *traffic density (in thousands)*. This variable was derived from the California Department of Public Health, the San Diego Association of Governments, and CalEnviroScreen 2.0. It represents the summed traffic volume within 150 m of a census tract boundary as of 2004. It is adjusted by road segment length in vehicle-kilometers per hour, divided by the total road length in kilometers, and put in thousands of units for ease of interpretation. The industrial emission sources were operationalized with the 2004 estimates of the *percent of industrial-zoned land* from the State of California general plan database. The general plans database was created by the California Resources Agency and the University of California, Davis [62]. To create this measure, the extent to which “heavy” and “light” industrial zoning polygons from the general plans database covered the area of 2010 census tract boundaries were calculated.

### 2.6. Spatial Factors

This study’s conceptual framework and the particularities of the California case highlight how spatial dynamics significantly influence the distribution of PM_2.5_ levels in ways that are not accounted for by other variables included in the analysis. In the analytic strategy section below, I discuss how I account for the local clustering of PM_2.5_ in this study. In this section, I summarize how I accounted for influential regional patterning of PM_2.5_.

California’s 15 air basins have unique meteorological, geographic, air pollution, and transport characteristics, which are recognized in California state statutes [63]. The prior study using CalEnviroScreen 2.0 found that PM_2.5_ is a major contributor to cumulative pollution burden in California [18] and also found that the extent of tract intersection with the South Coast air basin (Los Angeles, Orange, Riverside, San Bernardino, and Ventura Counties) and the San Joaquin Valley (SJV) air basin (Fresno, Kern, Kings, Madera, Merced, San Joaquin, Stanislaus, and Tulare Counties) were positive multivariable correlates of cumulative pollution burden [18]. Those same air basins contain the only counties in the United States that are expected to exceed the NAAQS for PM_2.5_ of 12 μm/m^3^ by 2020 [17]. Accordingly, this study used the spatial data on California’s air basins and created two regional correlates of PM_2.5_ levels: *the percent of tract in the South Coast air basin* and *the percent of tract in the SJV air basin*.

### 2.7. Analytical Strategy

The analysis presented below begins with a summary of the descriptive statistics for all variables used in this study including a visual display of PM_2.5_ variation across California census tracts over the 2009–2011 monitoring period. It follows with a summary of the bivariate correlations between all variables used in the multivariable regression analysis of PM_2.5_ percentile rankings in California.

After reviewing those univariate and bivariate patterns, standard spatial econometric techniques within the environmental inequality literature [18,59,64] were used to account for additional spatial effects in the regression analyses that were not accounted for by other analysis variables. This entails running the multivariable regression analysis of PM_2.5_ percentile rankings with initial ordinary least squares (OLS) regression models, and analyzing the residuals from those models with global Moran’s I tests for spatial autocorrelation using different spatial weights matrices. These tests showed that the OLS residuals exhibited significant and positive spatial autocorrelation. The Lagrange Multiplier (LM) diagnostic revealed a higher LM value for the spatial lag model over the spatial error model. Accordingly, the spatial lag specifications were used to account for spatial effects in the regression analyses [65]. Spatial lag models address potential biases in coefficient standard errors that are associated with multidirectional and simultaneous spatial dependence and endogeneity in the estimation of regression model parameters [66].

The spatial lag models are generally defined as:

$$y=\;\alpha +\rho Wy+\underset{k}{\sum }\beta_{k}X_{k}+u$$

where y represents percentiles of annual mean PM_2.5_ concentrations; α is the intercept; ρ is the spatial influence coefficient; *W*y is the spatially lagged dependent variable for the *W* spatial weights matrix; β is the coefficient for the *k* number of *X* population vulnerability, emission source, and air basin regional control variables; and *u* is the spatially independent error term [65,66].

The spatial weights matrix captures the spatial relationships between census tracts. Similar to the prior study of cumulative pollution burden with CalEnviroScreen 2.0 [18], it was found that a row-standardized first-order queen adjacency spatial weights matrix—when used with the regional controls for tract intersection with the South Coast and SJV air basins—successfully accounted for spatial dependence in the spatial lag models. The matrix resulted in only five tracts without a neighbor out of the 7533 tracts included in the regression analyses that had non-missing values for all variables. There were a maximum of 23 and mean of six neighbors across the remaining 99.93 percent (*N* = 7528) tracts with a neighbor. For consistency the same spatial weights matrix for all spatial autocorrelation tests was used in this study.

## 3. Results

### 3.1. Descriptive Statistics

**Figure 2:**
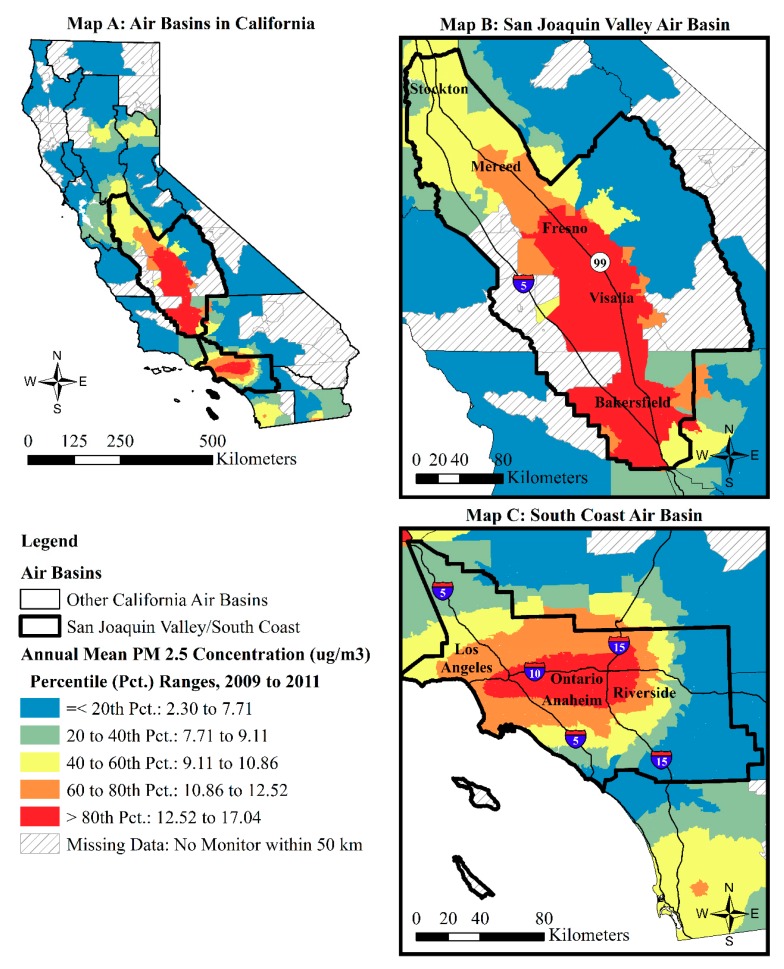
Percentile ranges of annual mean PM_2.5_ concentrations from 2009 to 2011 in (**A**) California, (**B**) the San Joaquin Valley air basin, and (**C**) the South Coast air basin.

Figure 2 shows the percentile ranges of the 2009–2011 annual mean PM_2.5_ concentrations for 7944 census tracts with non-missing data in California. The figure visualizes the elevated concentrations of PM_2.5_ in the SJV and South Coast air basins.

Of the 7944 census tracts shown in Figure 2, 2363 tracts (29.75 percent) had 2009–2011 annual PM_2.5_ concentrations that exceeded the 2012 NAAQS of 12 μm/m^3^. Those tracts ranked in the 71st percentile in the statewide distribution. The vast majority of those tracts intersect the South Coast air basin (*N* = 1944; 82.3 percent) and the SJV air basin (*N* = 420; 17.8 percent). There were only five other tracts that exceeded the PM_2.5_ NAAQS and intersected with other air basin boundaries: three (0.01 percent) intersected the Mojave Desert air basin and two (0.01 percent) intersected the South Central Coast air basin. The detail maps in Figure 2 appear consistent with research that demonstrates that traffic volume is a major source of elevated PM_2.5_ concentrations [2]. Map B is consistent with literature on the poor air quality of the SJV, particularly in its southern expanse that is characterized as a “bowl” and that retains much of the traffic (and industrial) emissions in the air, which contributes to asthma-related emergency department visits and other adverse respiratory conditions among residents [61]. Similarly, Map C coheres with prior studies that documented how high traffic volumes combine with the topography and meteorology of the South Coast to concentrate elevated PM_2.5_ levels from downtown Los Angeles to the San Bernardino corridor along Interstate 10 [24].

Table 3 displays the descriptive statistics for all variables used in the bivariate and spatial lag regression analyses with the final analytical sample of 7533 tracts. There are a couple noteworthy patterns in the table. First, the annual average PM_2.5_ had a mean percentile of 50.35, which corresponded to an annual average PM_2.5_ level below the NAAQS at 10.05 μm/m^3^. Second, there were both high degrees of spatial clustering (Moran’s I ≥ 0.830) and variance regarding the mean for percentiles of annual average PM_2.5_ concentration, the air basin regional controls, and the percent Latinx population. Percent non-Latinx Asian population and percent non-Latinx Black population were also highly clustered (Moran’s I = 0.817), but they had less variation regarding the mean. The bivariate correlations summarized below indicate some of the environmental inequality implications of how these variables are distributed throughout the California census tracts.

### 3.2. Bivariate Correlations

The results presented in Table A1 summarize the bivariate relationships between the PM_2.5_ percentile rankings, race-based population vulnerability factors, the CalEnviroScreen 2.0 population vulnerability factors, emission sources, and air basin regional controls. All of the independent variables had positive and significant bivariate correlations with the annual average PM_2.5_ percentile rankings, except for percent non-Latinx Asian population. Among the population vulnerability variables with a significant association, the annual average PM_2.5_ percentile ranking was most strongly correlated with the structural, race-based vulnerability factor of percent Latinx population, followed by isolated Latinx economic disadvantage, and isolated economic disadvantage—measures that are strongly associated with extrinsic vulnerability. In contrast, the other structural, race-based vulnerability factor of percent non-Latinx Black and the related intrinsic vulnerability measures of Black health disadvantage and health disadvantage had low bivariate correlations with annual average PM_2.5_ percentile rankings. These initial results illustrate (1) how various spatial dimensions of extrinsic and intrinsic vulnerability are differentially associated neighborhood racial composition and PM_2.5_ exposures, and (2) the existence of environmental racial inequality in the distribution of PM_2.5_ in California, particularly among tracts with elevated levels of Latinx residents.

Table A1 also helps to diagnose the degree of intercorrelation among the independent variables prior to assessing the multicollinearity diagnostics in the multivariable spatial lag regression models. The table shows that select population vulnerability variables have high intercorrelations (Pearson *r* ≥ 0.80). However, the spatial lag regression models discussed below mitigate against such collinearity by entering those highly correlated population vulnerability variables into separate regression models.

### 3.3. Multivariable Correlates of PM_2.5_ Exposure

Table 4 summarizes the spatial lag regression results for annual average PM_2.5_ percentile ranking. All models in the table account for the same emission source and spatial factors that influence the distribution of annual average PM_2.5_. The models vary according to their inclusion of different population vulnerability variables and the corresponding hypotheses they test. The multicollinearity condition indices (MCIs) for all models were all well below the suggested threshold of 30 [65]. These low MCIs indicate that the models are relatively parsimonious and this modeling approach avoids problems of serious multicollinearity among the independent variables. In addition, spatial dependence was not evident in these models, as indicated by their highly statistically significant spatial autoregressive coefficient (Rho), and the insignificant global Moran’s I values for the spatial lag regression residuals.

Model 1 uses the race-based, structural vulnerability factors of percent non-Latinx Asian population, percent non-Latinx Black population, and the percent Latinx population variables to test the Asian (H1), Black (H2), and Latinx (H3) environmental inequality hypotheses, respectively. Model 1 also accounts for the CalEnviroScreen extrinsic vulnerability factor of isolated economic disadvantage and intrinsic vulnerability factor of health disadvantage. Both of these factors were negatively correlated with the percentile rankings of PM_2.5_ in Model 1, most likely due to their moderate intercorrelation with the race-based, structural vulnerability variables of the percent non-Latinx Black population and percent Latinx population (see Table A1). All three race-based, structural vulnerability factors, however, were positively and significantly associated with annual average PM_2.5_ percentile rankings, net of other factors in the model. Those results support the Asian (H1), Black (H2), and Latinx (H3) environmental inequality hypotheses. The unstandardized coefficients (b) suggest that a one-point increase in percent non-Latinx Asian population, percent non-Latinx Black population, and percent Latinx population was associated with a 0.70-, 1.30-, and 1.10-percentile increase in the rankings of annual average PM_2.5_, respectively, net of other factors in Model 1.

Model 2 retains the percent non-Latinx Asian population and replaces the other population vulnerability variables in Model 1 with Black health disadvantage and isolated Latinx economic disadvantage. In so doing, Model 2 tests the Asian environmental inequality hypothesis (H1) as well as the Black (H4) and Latinx (H5) concentrated disadvantage hypotheses. The results in Model 2 show that only percent non-Latinx Asian and isolated Latinx economic disadvantage exhibited a positive and significant effect on the percentile ranking of PM_2.5_ in the spatial lag regression analysis. These results support the Asian environmental inequality hypothesis (H1) and the Latinx concentrated disadvantage (H5) hypothesis. A one-point increase in percent non-Latinx Asian population and in isolated Latinx economic disadvantage was significantly associated with a 0.30- and 9.0-percentile increase in the ranking of annual average PM_2.5_, respectively, net of other factors included in Model 2. In contrast, the coefficient for Black health disadvantage was not signed as expected, and was not significant. This result does not support the Black concentrated disadvantage (H4) hypothesis.

Models 1 and 2 unexpectedly displayed negative and null multivariable associations between the intrinsic vulnerability factors (health disadvantage and Black health disadvantage) and percentile rankings of annual average PM_2.5_. Given those results, Model 3 only included percent non-Latinx Asian population, percent non-Latinx Black population, and isolated Latinx economic disadvantage as population vulnerability factors for annual average PM_2.5_ exposure. The unstandardized coefficients for these variables were all significant and positively signed as expected, lending support for the Asian (H1) and Black (H2) environmental inequality hypotheses as well as for the Latinx concentrated disadvantage hypothesis (H5). Net of the other factors included in Model 3, a one-point increase in percent non-Latinx Asian population, in percent non-Latinx Black population, and in isolated Latinx economic disadvantage was significantly associated with a 0.40-, 0.50-, and 9.7-percentile increase in the ranking of annual average PM_2.5_, respectively.

Of the other variables included in the spatial lag models, the air basin regional controls and the spatially lagged percentile ranking of annual average PM_2.5_ consistently performed as expected. That is, in the three models, these spatial factors were positively associated with the percentile ranking of annual average PM_2.5_. The emission sources variables exhibited no significant effect on the percentile ranking of annual average PM_2.5_ in Model 1. However, traffic density had a significant and positive effect as expected on the percentile ranking of annual average PM_2.5_ in Model 2 and approached a significant (*p* = 0.055) and positive effect on the annual average PM_2.5_ percentile rankings in Model 3.

Additional results presented in Table 4 helped to compare and contrast the models and the effects of their independent variables. Smaller Akaike information criterion and larger log likelihood statistics indicate an improved fit when comparing competing models [65,66]. The unit-less standardized regression coefficient (B) aids in assessing the relative strength of the independent variables in each model. Higher standardized coefficients indicate stronger effects of an independent variable on the percentile ranking of annual average PM_2.5_. Using these criteria, we can first conclude that Model 1 fits the data better than Models 2 and 3. Second, the standardized coefficients in Model 1 suggest that the percent Latinx population, followed by the regional clustering of PM_2.5_, and the percent of non-Latinx Black and non-Latinx Asian population were the strongest positive multivariable correlates of PM_2.5_ percentile rankings, net of the other factors included in the analysis.

## 4. Discussion

PM_2.5_ is a major environmental health hazard, particularly in the United States for Asian, Black, and Latinx populations. Airborne PM_2.5_ concentrations exceed national standards, particularly in the South Coast and San Joaquin Valley air basins where a significant number of Asian, Black, Latinx, and other racial minorities predominate. Despite the environmental health and inequality implications of these conditions, previous research on racial disparities in PM_2.5_ exposures throughout California is limited and at times inconsistent with nationwide studies.

The present study addresses these limitations in prior research on PM_2.5_ by adapting the conceptual framework and analytical techniques previously used in Liévanos [18] to “retool” CalEnviroScreen 2.0 and illuminate the race-based environmental health vulnerabilities associated with cumulative pollution burden. That study found that PM_2.5_ was the major environmental health hazard contributor to cumulative pollution burden in California. It also found that the tracts’ elevated levels of Black and Latinx composition were each positively correlated with the cumulative pollution burden. Furthermore, its regression analysis found that the tract Latinx composition was the strongest net demographic multivariable correlate of cumulative pollution burden.

While insightful, Liévanos [18] also had important limitations. First, it did not assess the bivariate and multivariable relationship between population vulnerability and PM_2.5_, the major environmental health hazard contributor to cumulative pollution burden in California. Second, it conceptualized neighborhood racial composition as an extrinsic dimension of environmental health vulnerability in accordance with prior environmental health scholarship [28]. Accordingly, it undertheorized how neighborhood racial composition is a “structural,” community-level [9] component of population vulnerability of environmental health hazard exposure that is arguably distinct from extrinsic factors of socioeconomic status and intrinsic factors of population sensitivity and susceptibility. Third, it did not analyze Asian environmental inequality outcomes because of its reliance on previous literature and the empirical details of the California case, which collectively indicated that tract-level Black and Latinx composition were the consistent race-based population vulnerability correlates of cumulative pollution burden.

This present study addressed the limitations in Liévanos [18] and related statewide research on California [19,20,21,58] through the following. First, it drew on the perspectives of critical race, space, and environmental justice studies [7,8,9,29,30,31,32,33,34,35,36,37,38,39,40,41,42,43,44,45,46,47,48,49,50,51], and empirical research on select regions of California and throughout the United States [7,8,9,10,11,12,13,14,15,22,23,24,25,26,27,34,38,39,52,53,54,55,56,57,59] to advance a “racialized structural vulnerability” approach to environmental health vulnerability, particularly for Asians, Blacks, and Latinxs, that is separate from extrinsic and intrinsic vulnerability. This framework elaborates on the community-level component of Gee and Payne-Sturges’s [9] environmental health disparities framework. It bases the analysis of race-based environmental health vulnerabilities within a contemporary environmental racial formation that has historical roots in California [34,41,42,43,44,45,47,49,52,56]. This environmental racial formation upholds the elite status and environmental privilege of Whites (e.g., low PM_2.5_ exposures) through a variety of institutional mechanisms that maintain racial segregation and lead to the relative subordination and heightened environmental health hazard (e.g., PM_2.5_) exposures of Asian, Black, and Latinx bodies and spaces. The racialized structural vulnerability perspective thus argues against subsuming neighborhood racial composition within the realm of “extrinsic” vulnerability factors like socioeconomic status [28] (p. 85). It especially argues against excluding neighborhood racial composition all together from the data as is done with colorblind tools like CalEnviroScreen [20,21,58]. Such omissions grossly miss the “social facts” [42] and political projects [43] of race, racism, and environmental racial inequality, especially in California [14,18,22,23,24,25,26,27,34,38,39,41,42,43,44,45,49,51,52,53,59].

Empirically, this study showed that tract-level Latinx, Black, and Asian composition were among the strongest positive multivariable correlates of PM_2.5_ percentile rankings in California, net of other factors (see Table 4, Model 1). A secondary finding of this study is that it demonstrated that tract-level Latinx and Black composition were significantly and differentially associated with concentrated socioeconomic and health disadvantage, respectively, as well as with elevated PM_2.5_ exposures for Black and Latinx populations in California (see Table 1 and Table A1) in ways that have not been uncovered in previous research [18].

This secondary finding was facilitated by the development of two new population vulnerability indicators of “concentrated disadvantage”—isolated Latinx economic disadvantage and Black health disadvantage—with a single PCA approach that illuminated how extrinsic and intrinsic vulnerability were differentially associated with the structural vulnerability factor of neighborhood racial composition. These new factors improve upon prior related research [18,20] by capturing how population vulnerabilities operate spatially for different racial groups in California. The isolated Latinx economic disadvantage factor variable is consistent with prior research on Latinx environmental inequalities, which shows how economic disadvantage and linguistic isolation (indicators of extrinsic vulnerability) are concentrated among segregated Latinx residents [10,11,14,18,59]. The Black health disadvantage factor variable is consistent with California-based environmental and public health research, which shows that segregated Black residents have an elevated prevalence of asthma and low birth weight (indicators of intrinsic vulnerability) [52,53]. In addition, the present study found these race-based, concentrated disadvantage factor variables performed differently in the multivariable regression analyses when compared to models that included the tract racial composition variables alongside the other new factor variables developed in this study: isolated economic disadvantage (a measure of extrinsic vulnerability) and health disadvantage (a measure of intrinsic vulnerability) (see Table 4, Models 1 and 2).

In sum, this study demonstrates how neighborhood racial composition is significantly associated with concentrated disadvantage and PM_2.5_ exposure vulnerabilities differentially among Asians, Blacks, and Latinxs in ways that elaborate on similar findings regarding cumulative pollution burden in California during the same period [18]. An important emergent theme across all the results of this study is that tracts with elevated concentrations of Latinxs are particularly double-burdened by elevated levels of extrinsic vulnerability and PM_2.5_ exposures in California, net of other factors considered in the analysis.

### Limitations and Future Research and Policy Implications

Despite its contributions, future research should address important limitations of this study. First, the main analyses in this study used data from 2004 to 2012, and especially from CalEnviroScreen 2.0, to examine the effects of neighborhood racial composition, other indicators of population vulnerability, traffic and industrial emission sources, and spatial dynamics on PM_2.5_ exposures in California. To what extent do the results differ when using more recent data from CalEnviroScreen 3.0? The Appendix A presents additional bivariate correlation analyses that consider the main variables used in this study, and the percentile rankings of annual average PM_2.5_ concentrations from 2012–2014 and the change in percentile rankings of annual average PM_2.5_ concentrations from 2009–2011 to 2012–2014. The annual average PM_2.5_ concentration estimates for 2012–2014 were derived from CalEnviroScreen 3.0. These were similarly measured as the 2009–2011 period in CalEnviroScreen 2.0 [58,67,68]. In briefly discussing these additional analyses, this section seeks to draw out future research and policy implications from this study.

Table A2 shows that the percentile rankings of annual average PM_2.5_ concentrations from 2009–2011 were positively, but not perfectly, associated with the percentile rankings of annual average PM_2.5_ concentrations from 2012–2014 (Pearson *r* = 0.902; *p* < 0.001). This association as well as the minor negative correlation between the percentile rankings of the annual average PM_2.5_ concentrations from 2009–2011 and change in the percentile rankings of annual average PM_2.5_ concentrations from 2009–2011 to 2012–2014, indicates that there have been some minor reductions in PM_2.5_ concentrations across California census tracts following the establishment of the new PM_2.5_ NAAQS of 12 μm/m^3^ in 2012. However, Table A2 also suggests that those reductions in annual average PM_2.5_ concentrations are more consistently associated with tract-level Asian composition, but not so for tracts with elevated levels of Blacks and Latinxs, wherein annual average PM_2.5_ concentrations remained significantly high in 2012–2014. In addition, percentile rankings of annual average PM_2.5_ concentrations from 2012–2014 were positively associated with this study’s factor variables of health disadvantage, isolated economic disadvantage, Black health disadvantage, and isolated Latinx economic disadvantage as well as with industrial zoning and traffic density in 2004 and tract containment in the South Coast and SJV air basins. Of these, the change in percentile rankings of annual average PM_2.5_ concentrations from 2009–2011 to 2012–2014 was positively associated with the factor variables and percent tract containment in the SJV air basin. The change in percentile rankings of PM_2.5_ were negatively associated with the tract containment in the South Coast air basin.

There are important policy implications from the main findings of the present study as well as the preliminary demographic and spatial relationships summarized in Table A2. Namely, they speak to the urgent need for targeted PM_2.5_ reduction strategies for the SJV and its vulnerable neighborhoods experiencing concentrated health and socioeconomic disadvantages. Decades of environmental justice organizing and research by allied environmental health scientists throughout the SJV have helped to bring increased state-level regulatory scrutiny to the inadequate and exclusionary development patterns, environmental policies, and decision making processes in the SJV. Advocates and allies have targeted these developments, policies, and processes because they have placed vulnerable residents and communities at increased risk of exposures to toxic air pollution and other hazardous substances in the SJV [69,70,71,72,73,74,75,76,77,78,79].

Compelled by these environmental justice movement and allied efforts, the California Air Resources Board (CARB) and the San Joaquin Valley Air Pollution Control District (SJVAPCD) produced a new “2018 Valley PM_2.5_ Plan” that was approved by the US EPA [80,81]. The plan moves the SJV to the “Serious” nonattainment classification for four major federal PM_2.5_ standards, particularly the 2012 annual NAAQS of 12 μm/m^3^, which mobilizes additional resources and institutional mechanisms to move the SJV into the attainment of those federal standards in the 2020s. In addition, it is claimed by officials to be a precedent-setting, integrated approach to aggressively reducing primary and secondary contributors to PM_2.5_ in the SJV through combining old and new regulatory controls and voluntary incentives from the CARB that address mobile sources and from the SJVAPCD, which address stationary and area sources [80,81]. The SJVAPCD prepared a “Negative Declaration for the 2018 PM_2.5_ Plan,” that found that the plan “would not have a significant impact on air quality and would have a less than significant impact on the environment” [80] (p. 28).

In keeping with a continued focus on “participatory justice” within the Californian environmental protection agencies [72,74,75,76], considerable efforts were apparently made by the CARB to improve public participation in and deliberation about the plan [80]. However, it remains to be seen how participating agencies in the plan assessed or plan to monitor the distributional effects and environmental health equity implications of the plan. Nor does the plan stipulate how it may achieve its goals in coordination with California’s broader effort to combat the dual problems of cumulative pollution burden and climate change vulnerabilities [18,75,82], which have been previously identified as significantly concentrated in the SJV [70].

There are significant political and organizational constraints on regulatory scientists and officials to pursue such questions in California [18,72,74,75,76,83] and under the Trump Administration’s US EPA [11,84]. Therefore, it is incumbent upon advocates and academic scientists to conduct critical analyses of the environmental health equity implications of any PM_2.5_-related policies and proposed reduction strategies in California. This study’s analytical techniques and racialized structural vulnerability framework could guide future advocacy, environmental, public health, and social science research aimed at taking on this arduous task. In addition, there are a number of nationwide datasets that were not featured in this study that could inform such analyses at both the census tract and census block group levels. These other datasets could include updated population, housing, and land use estimates from the Decennial Census, the ACS, and the US Geological Survey’s National Land Cover Database (NLCD) [10,11,59], respectively. They can also include more frequent estimates of traffic volume and PM_2.5_ exposures from the US EPA’s Community Multi-Scale Air Quality Model [12], the US EPA’s National Emissions Inventory (NEI) [85,86], and the US EPA’s EJSCREEN [87,88,89,90]. When merged together, particularly at the census block group level, these data can provide a higher-resolution analysis of race-based ambient PM_2.5_ exposure vulnerabilities, and their relationship to recent estimates of industrial land use and traffic volume in California. Assessing such relationships with regularly-updated data and at a finer scale could overcome this study’s use of the 2004 industrial zoning and traffic volume estimates, which performed poorly in the regression analyses at the census tract level, despite the known relationship between industrial and traffic emissions and PM_2.5_ [2].

In addition, future research could pair the alternative nationwide datasets listed above with other data that can advance our understanding of race-based PM_2.5_ exposure vulnerabilities and exposures to facility-level “co-pollutants” of PM_2.5_ and CO_2_ in California [75,85]. This additional data includes, in particular, facility-level carbon dioxide (CO_2_) emissions estimates from the US EPA Greenhouse Gas Reporting Program [85] and California’s Climate Investments (CCI) Project Map [90]. The CCI Project Map is associated with California’s program to redistribute Greenhouse Gas Reduction Funds from its carbon cap-and-trade program to the most overburdened census tracts identified with CalEnviroScreen data [18,75,82].

Furthermore, this study acknowledged the important historical and institutional forces that generally contribute to the existence of race-based environmental health vulnerabilities in California. However, it did not directly measure the effects of those forces on contemporary PM_2.5_ exposure vulnerabilities nor on the health outcomes of such exposure. To do so, future research could link the racialized structural vulnerability framework, drawn in part from critical race, space, and environmental justice perspectives [9,29,30,31,32,33,34,35,36,37,38,39,40,41,42,43,44,45,46,47,48,49,50,51], with Sewell’s [91] “racism-race reification process” model of racial health disparities. The resulting synthesis could examine, for example, how racial disparities in asthma hospitalizations in California [92] are associated with contemporary racial inequalities in mortgage market access (as identified with Home Mortgage Disclosure Act data) and PM_2.5_ exposures as well as historical patterns of racial and spatial exclusion from the home mortgage market (i.e., “redlining”) [44,48,49,50]. Illuminating the influence of such historical and institutional forces might help to identify causal factors in the formation of race-based environmental health vulnerabilities as well as strategic points of intervention in planning and governance processes to advance environmental justice and health equity [93].

## 5. Conclusions

This statewide, tract-level, cross-sectional study contributes to previous research by advancing a racialized structural vulnerability framework for examining the relationship between neighborhood racial composition, intrinsic and extrinsic vulnerability, and PM_2.5_ exposures in California. It generally supports the contention that racial segregation is a distinct “structural,” community-level factor [9] within the realm of population vulnerability and environmental health disparities. Using principal component analyses, it found that the tract Latinx composition was highly correlated with extrinsic vulnerability (economic disadvantage and limited English-speaking ability), and that tract Black composition was highly correlated with intrinsic vulnerability (elevated prevalence of asthma-related emergency department visits and low birth weight). The results from spatial lag regression analyses supported hypotheses that posited a positive association between the neighborhoods’ Asian, Black, and Latinx composition and 2009–2011 annual average PM_2.5_ percentile rankings, net of other population vulnerability, emissions, and spatial covariates. Its primary finding was that neighborhoods with higher shares of Latinxs appear to be particularly double-burdened by elevated levels of extrinsic vulnerability and PM_2.5_ exposures in California, net of other factors considered in the analysis. This particular finding supports prior research regarding socioeconomic inequalities and environmental health disparities experienced by Latinxs in the United States [10,11,13,54]. When understood in the context of previous research in California [18], the present study provides more evidence that the spatial concentration of Nonwhites, particularly of Latinx people, is emerging as a primary multivariable correlate of residential exposure to cumulative pollution burden and one of its main constituent elements: PM_2.5_. However, these environmental racial inequalities are not evident when researchers and policy makers do not consider race-based environmental health vulnerabilities in their analyses when subsuming “race” into broad extrinsic vulnerability factors [28] or due to the preference for colorblind environmental health hazard screening tools like CalEnviroScreen. Instead, race and space matter in structuring environmental health vulnerabilities and the pursuit of environmental health equity. Indeed, a clear implication of this study is that addressing cumulative impacts and achieving environmental health equity in California will rely on (1) recognizing the continuing significance of neighborhood racial composition as a powerful and independent environmental health vulnerability factor and (2) significantly lowering PM_2.5_ levels *and* racial segregation of Latinxs, Blacks, and Asians in the state.

## Figures and Tables

**Table 1:** Principal component analysis results for race-based concentrated disadvantage (*N* = 7610 tracts) ^1^.

Variable	Component Factor Loadings	
	1: Isolated Latinx Economic Disadvantage	2: Black Health Disadvantage
Percent Latinx population, 2010	0.891	0.101
Percent non-Latinx Black population, 2010	−0.078	0.846
Average percent linguistically isolated households, 2008–2012	0.841	−0.049
Average percent of population over 25 with less than a high school education, 2008–2012	0.945	0.180
Average percent of population living below twice the federal poverty level, 2008–2012	0.844	0.351
Average percent of the population over the age of 16 that is unemployed, 2008–2012	0.483	0.466
Age-adjusted asthma-related emergency department visits, 2007–2009	0.247	0.791
Percent low-weight births (LWB), 2006–2009	0.123	0.610
*Component diagnostics*		
Eigenvalue	3.421	2.098
Percent of total variance explained	42.76	26.23

^1^ Principal components were extracted using varimax rotation and eigenvalues greater than 1. Cumulative total variance explained by both components: 68.99%.

**Table 2:** Principal component analysis results for CalEnviroScreen 2.0 extrinsic and intrinsic vulnerability indicators (*N* = 7610 tracts) ^1^.

Variable	Component Factor Loadings	
	1: Isolated Economic Disadvantage	2: Health Disadvantage
Average percent linguistically isolated households, 2008–2012	0.908	−0.040
Average percent of population over 25 with less than a high school education, 2008–2012	0.904	0.268
Average percent of population living below twice the federal poverty level, 2008–2012	0.823	0.430
Average percent of the population over the age of 16 that is unemployed, 2008–2012	0.409	0.610
Age-adjusted asthma-related emergency department visits, 2007–2009	0.179	0.793
Percent low-weight births (LWB), 2006–2009	0.015	0.699
*Component diagnostics*		
Eigenvalue	3.169	1.098
Percent of total variance explained	52.81	18.29

^1^ Principal components were extracted using varimax rotation and eigenvalues greater than 1. Cumulative total variance explained by both components: 71.10%.

**Table 3:** Descriptive statistics of variables used in the bivariate correlation and spatial lag regression analyses (*N* = 7533 tracts).

Variable	Mean	S.D.	Min.	Max.	Moran’s I ^2^
Percentile of annual average PM_2.5_ concentration, 2009–2011 ^1^	50.35	28.79	0.01	100.00	0.992 ***
Percent non-Latinx Asian population, 2010	12.78	14.83	0.00	89.81	0.817 ***
Percent non-Latinx Black population, 2010	5.83	9.33	0.00	89.76	0.817 ***
Percent Latinx population, 2010	37.03	26.43	1.19	99.03	0.830 ***
Health disadvantage, 2006–2012	0.00	1.00	−3.13	4.96	0.657 ***
Isolated economic disadvantage, 2006–2012	0.00	1.00	−1.88	4.92	0.687 ***
Black health disadvantage, 2006–2012	0.00	1.00	−2.14	6.54	0.784 ***
Isolated Latinx economic disadvantage, 2006–2012	0.00	1.00	−2.66	3.61	0.752 ***
Percent of industrial-zoned land, 2004	4.80	12.13	0.00	93.73	0.297 ***
Traffic density (1000 s), 2004	1.27	1.22	0.00	43.56	0.462 ***
Percent tract in the South Coast	43.83	49.59	0.00	100.00	0.995 ***
Percent tract in the San Joaquin Valley	9.14	28.78	0.00	100.00	0.990 ***

^1^ PM_2.5_: particulate matter less than 2.5 μm. ^2^ Moran’s I analysis used a first-order queens adjacency spatial weights matrix. *** Correlation is significant at the 0.001 level (two-tailed; 9999 permutations).

**Table 4:** Spatial lag regression results for percentile of Annual Average PM_2.5_ concentrations, 2009–2011 (*N* = 7533 tracts) ^1^.

Variable	Model 1			Model 2			Model 3		
	b ^2^	S.E. ^3^	B ^4^	b	S.E.	B	b	S.E.	B
*Population vulnerability*									
Percent non-Latinx Asian population, 2010	0.007 ***	0.002	0.004	0.003 *	0.002	0.002	0.004 *	0.002	0.002
Percent non-Latinx Black population, 2010	0.013 ***	0.003	0.004				0.005 *	0.003	0.002
Percent Latinx population, 2010	0.011 ***	0.002	0.010						
Health disadvantage, 2006–2012	−0.142 ***	0.033	−0.005						
Isolated economic disadvantage, 2006–2012	−0.129 **	0.043	−0.004						
Black health disadvantage, 2006–2012				0.003	0.024	0.000			
Isolated Latinx economic disadvantage, 2006–2012				0.090 ***	0.026	0.003	0.097 ***	0.026	0.003
*Emission sources*									
Percent of industrial-zoned land, 2004	0.001	0.002	0.000	0.001	0.002	0.000	0.001	0.002	0.000
Traffic density (1000 s), 2004	0.028	0.020	0.001	0.040 *	0.020	0.002	0.039 ^†^	0.020	0.001
*Air basin*									
Percent tract in the South Coast	0.004 ***	0.001	0.007	0.005 ***	0.001	0.008	0.004 ***	0.001	0.008
Percent tract in the San Joaquin Valley	0.009 ***	0.001	0.009	0.008 ***	0.001	0.008	0.009 ***	0.001	0.009
Rho	0.986 ***	0.001		0.987 ***	0.001		0.987 ***	0.001	
(Intercept)	−0.178 *	0.086		0.264 ***	0.059		0.241 ***	0.060	
*Model diagnostics*									
Multicollinearity condition index	10.139			4.100			4.400		
Pseudo R ^2^	0.995			0.995			0.995		
Log likelihood	−17,531.40			−17,554.60			−17,552.50		
Degrees of freedom	7522.00			7524.00			7524.00		
Akaike information criterion	35084.80			35,127.20			35,123.00		
Moran’s I of residuals ^5^	−0.005			−0.006			−0.006		

^1^ PM_2.5_: particulate matter less than 2.5 μm; ^2^ b: unstandardized regression coefficient; ^3^ S.E.: standard error of the unstandardized regression coefficient; ^4^ B: standardized regression coefficient. ^5^ Moran’s I results are based on first-order queen adjacency spatial weights matrix and 9999 permutations. ^†^ Correlation is significant at the 0.10 level (two-tailed). * Correlation is significant at the 0.05 level (two-tailed). ** Correlation is significant at the 0.01 level (two-tailed). *** Correlation is significant at the 0.001 level (two-tailed).

## Appendix A

**Table A1:** Pearson correlation coefficients for variables used in the spatial lag regression analyses (N = 7533 census tracts).

Variable	1	2	3	4	5	6	7	8	9	10	11
1. Percentile of annual average PM_2.5_ concentrations, 2009–2011 ^1^											
2. Percent non-Latinx Asian population, 2010	0.02										
3. Percent non-Latinx Black population, 2010	0.10 ***	−0.07 ***									
4. Percent Latinx population, 2010	0.41 ***	−0.31 ***	0.04 ***								
5. Health disadvantage, 2006–2012	0.05 ***	−0.22 ***	0.56 ***	0.22 ***							
6. Isolated economic disadvantage, 2006–2012	0.30 ***	−0.04 **	−0.02 *	0.78 ***	0.01						
7. Black health disadvantage, 2006–2012	0.05 ***	−0.15 ***	0.85 ***	0.10 ***	0.91 ***	−0.05 ***					
8. Isolated Latinx economic disadvantage, 2006–2012	0.33 ***	−0.14 ***	−0.08 ***	0.89 ***	0.14 ***	0.96 ***	0.00				
9. Percent of industrial-zoned land, 2004	0.15 ***	−0.02	0.04 ***	0.22 ***	0.09 ***	0.18 ***	0.07 ***	0.20 ***			
10. Traffic density (1000 s), 2004	0.13 ***	0.14 ***	0.05 ***	0.06 ***	−0.05 ***	0.06 ***	−0.01	0.04 ***	0.11 ***		
11. Percent tract in the South Coast	0.59 ***	0.013	0.08 ***	0.28 ***	−0.09 ***	0.21 ***	−0.04 ***	0.21 ***	0.12 ***	0.17 ***	
12. Percent tract in the San Joaquin Valley	0.28 ***	−0.13 ***	−0.05 ***	0.14 ***	0.19 ***	0.10 ***	0.09 ***	0.15 ***	0.02	−0.15 ***	−0.28 ***

^1^ PM_2.5_: particulate matter less than 2.5 μm. * Correlation is significant at the 0.05 level (two-tailed). *** Correlation is significant at the 0.001 level (two-tailed).

**Table A2:** Pearson correlation coefficients measuring associations between percentile rankings of annual average PM_2.5_ concentrations in 2012–2014 and change in percentile rankings of annual average PM_2.5_ concentrations from 2009–2011 to 2012–2014 and variables included in the spatial lag regression analyses.

Variable	Percentile of Annual Average PM_2.5_ Concentration, 2012–2014 ^1^	Change in Percentile of Annual Average PM_2.5_ Concentration, 2009–2011 to 2012–2014
Percentile of annual average PM _2.5_ concentration, 2009–2011 ^1^	0.902 ***	−0.197 ***
N	7929	7929
*Population vulnerability*		
Percent non-Latinx Asian population, 2010	0.017	−0.031 **
Percent non-Latinx Black population, 2010	0.109 ***	0.016
Percent Latinx population, 2010	0.413 ***	0.009
N	8005	7918
Health disadvantage, 2006–2012	0.059 ***	0.049 ***
Isolated economic disadvantage, 2006–2012	0.331 ***	0.065 ***
Black health disadvantage, 2006–2012	0.062 ***	0.038 ***
Isolated Latinx economic disadvantage, 2006–2012	0.356 ***	0.056 ***
*N*	7593	7519
*Emission sources*		
Percent of industrial-zoned land, 2004	0.138 ***	−0.017
Traffic density (1000 s), 2004	0.133 ***	−0.016
*N*	8016	7929
*Air basin*		
Percent tract in the South Coast	0.513 ***	−0.163 ***
Percent tract in the San Joaquin Valley	0.411 ***	0.295 ***
*N*	8016	7929

^1^ PM_2.5_: particulate matter less than 2.5 μm. ** Correlation is significant at the 0.01 level (two-tailed). *** Correlation is significant at the 0.001 level (two-tailed).
